# PEAR: a fast and accurate Illumina Paired-End reAd mergeR

**DOI:** 10.1093/bioinformatics/btt593

**Published:** 2013-10-18

**Authors:** Jiajie Zhang, Kassian Kobert, Tomáš Flouri, Alexandros Stamatakis

**Affiliations:** ^1^The Exelixis Lab, Scientific Computing Group, Heidelberg Institute for Theoretical Studies, Schloss-Wolfsbrunnenweg 35, D-69118 Heidelberg, ^2^Graduate School for Computing in Medicine and Life Sciences, ^3^Institut für Neuro- und Bioinformatik, University of Lübeck, 23538 Lübeck and ^4^Karlsruhe Institute of Technology, Institute for Theoretical Informatics, Postfach 6980, 76128 Karlsruhe, Germany

## Abstract

**Motivation:** The Illumina paired-end sequencing technology can generate reads from both ends of target DNA fragments, which can subsequently be merged to increase the overall read length. There already exist tools for merging these paired-end reads when the target fragments are equally long. However, when fragment lengths vary and, in particular, when either the fragment size is shorter than a single-end read, or longer than twice the size of a single-end read, most state-of-the-art mergers fail to generate reliable results. Therefore, a robust tool is needed to merge paired-end reads that exhibit varying overlap lengths because of varying target fragment lengths.

**Results:** We present the PEAR software for merging raw Illumina paired-end reads from target fragments of varying length. The program evaluates all possible paired-end read overlaps and does not require the target fragment size as input. It also implements a statistical test for minimizing false-positive results. Tests on simulated *and* empirical data show that PEAR consistently generates highly accurate merged paired-end reads. A highly optimized implementation allows for merging millions of paired-end reads within a few minutes on a standard desktop computer. On multi-core architectures, the parallel version of PEAR shows linear speedups compared with the sequential version of PEAR.

**Availability and implementation:** PEAR is implemented in C and uses POSIX threads. It is freely available at http://www.exelixis-lab.org/web/software/pear.

**Contact:** Tomas.Flouri@h-its.org

## 1 INTRODUCTION

The Illumina sequencing platform can produce millions of short reads in a single run. The deep sequencing capability and low cost of the sequencing-by-synthesis technology is useful for a plethora of applications ranging from whole-genome sequencing (Liu *et al.*, 2012; Wang *et al.*, 2010) to profiling microbial communities by sequencing the hypervariable regions of the 16S ribosomal RNA (rRNA) gene (Bartram *et al.*, 2011; Caporaso *et al.*, 2011; Degnan and Ochman, 2012; Rodrigue *et al.*, 2010; Zhou *et al.*, 2011). However, single-end reads produced by the Illumina platform typically have a length that ranges from 75 to 300-bp. Furthermore, there is an exponential increase in error rates (ERs) along the reads (Cox *et al.*, 2010). The Illumina platform can also generate paired-end reads by sequencing the forward and reverse strands of each target DNA fragment. If the target DNA fragment size is smaller than twice the length of the single-end reads, that is, if there exists an overlap, the corresponding paired-end reads can be merged into a fragment. By merging paired-end reads, the overlapping region between them can also be deployed for correcting sequencing errors and potentially yield sequences of higher quality. Merging paired-end reads is the first processing step in a plethora of sequence analysis pipelines. Hence, its accuracy is crucial for all downstream analyses.

There exist several proof-of-concept mergers such as iTag (Degnan and Ochman, 2012), BIPES (Zhou *et al.*, 2011) and Shera (Rodrigue *et al.*, 2010). Some production-level mergers such as FLASH (Magoč and Salzberg, 2011), PANDAseq (Masella *et al.*, 2012) and COPE (Liu *et al.*, 2012) have also been recently introduced.

Shera merges the reads by maximizing the number of matches between the paired-end reads. Both, Shera and FLASH (see later in the text) ignore the quality scores of the base calls. Shera merges all reads and leaves it to the user to decide which merged reads are correct. Because it is a proof-of-concept implementation, it is up to 100 times slower than competing mergers.

FLASH constructs merged reads that maximize the overlap length-to-matches ratio. FLASH requires the mean DNA fragment size and standard deviation of the fragment size as input parameters. Therefore, it can only merge paired-end reads into fragments of ‘almost’ identical size. Furthermore, our tests show that FLASH performs poorly when the overlaps between reads are short (Section 3).

COPE deploys an analogous approach as FLASH for finding the best overlap, but also takes into account the quality scores of mismatches. COPE is designed to handle deep genome sequencing datasets. Thus, it considers that *k*-mers that occur infrequently are likely to be sequencing errors. COPE exhibits high memory requirements and also relatively long execution times.

PANDAseq merges fragments by maximizing the probability of true sequence matches, given the observed sequences. It combines quality scores with sequence matches and thereby improves merging quality. In contrast to FLASH, PANDAseq works well with short overlap regions and does not require prior knowledge of the target DNA fragment size. However, it assumes that all paired-end reads can be merged. Thus, if the sample contains DNA fragments that are at least twice as long as the single-end reads, PANDAseq exhibits a high false-positive rate (FPR).

**Fig. 1.**
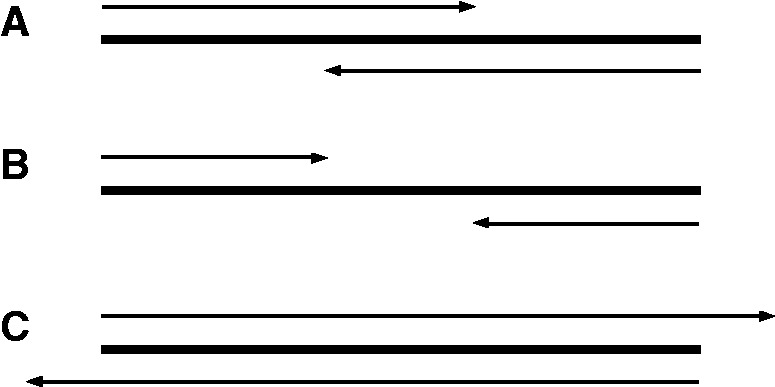
Three possible scenarios for paired-end read lengths and target DNA fragment lengths. (**A**) Short overlap between the paired-end reads; (**B**) no overlap between the paired-end reads; (**C**) single-end read length is larger than the target DNA fragment length

Finally, most current paired-end mergers assume that the DNA fragments are longer than the individual single-end reads. When this does not hold, for example when sequencing the V6 region of 16S rRNA genes of bacterial samples [fragment sizes range between 110 and 130-bp (Gloor *et al.*, 2010)] with read lengths of 150-bp (see case C in Fig. 1), current mergers will generate erroneous results.

Here we present PEAR, a fast and accurate paired-end read merger. PEAR merges reads by maximizing the *assembly score* (AS) of the read overlap via a scoring matrix that penalizes mismatches with a negative value β and rewards matches with a positive value α. Our approach takes quality scores *and* sequence matches into account. It does not require preprocessing of the raw data or specifying the fragment size. Furthermore, PEAR neither requires prior information on read length nor target fragment size. It can reliably identify reads that can either be merged or need to be discarded. The program is accurate on datasets with (i) short overlaps and (ii) DNA target fragment sizes that are smaller than single-end read lengths.

To identify false-positive merged reads, we propose a statistical test that is based on the observed expected alignment scores (OESs). On simulated paired-end reads with a mean overlap of 20-bp (Section 3.1), PEAR correctly merges 90.44% of the fragments with a FPR of 2.78% when our statistical test is disabled. It correctly merges 70.06% of the fragments with a FPR of only 0.48% when the significance level of the test is set to 1%. The best competing merger (PANDAseq) correctly merges 83.51% of the fragments, but with a FPR of 6.65%.

We implemented PEAR in C. It includes an optimized memory management scheme that allows the user to specify the amount of random access memory (RAM) available for executing the program. Therefore, it can be deployed on off-the-shelf desktop and laptop computers as well as on high-end multi-core servers. In Section 2.4 we outline why PEAR becomes faster when using less memory. Finally, the parallel version of PEAR scales linearly with the number of cores.

## 2 IMPLEMENTATION

In paired-end sequencing mode, the Illumina *Consensus Assessment of Sequence and Variation* (CASAVA) software generates two FASTQ files (Cock *et al.*, 2010), one for each reading direction of the fragment. The files contain exactly the same number of reads. Corresponding paired-end reads can be identified by their coordinates in the flow cell. The Illumina flow cell is a planar optically transparent surface similar to a microscope slide. It contains a lawn of oligonucleotide anchors bound to its surface.

PEAR scores all possible overlaps for each pair of corresponding paired-end reads to determine the overlap with the highest AS. Subsequently, PEAR conducts a statistical test to assess the statistical significance of the merged reads. If the merged reads do not pass this test or if the overlap length is smaller than a user-defined threshold (based on the expected approximate sequence length in the experiment) the pair of reads will not be merged. Otherwise, PEAR returns the merged fragment and will also correct errors using the Illumina quality scores.

### 2.1 Overlap algorithm

For each base, CASAVA (v1.8) yields an ASCII-encoded quality score that represents an integer value *Q*, which can be converted into the probability *e* of a sequencing error at the base via 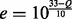 (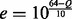 in earlier CASAVA versions). The base frequency *b* of a nucleotide is the number of occurrences of that nucleotide in the FASTQ files divided by the total number of bases. The probability *q* of a random base match is 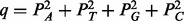. Given an overlapping region 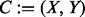, where *X* and *Y* are the overlapping segments of the two reads, we denote the observed (respectively true) base at position *i* of the overlap by 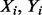 (respectively 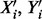). We denote the length of the overlap region by 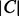. The probability that base *X_i_* (resp. *Y_i_*) is erroneous is 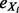 (resp. 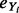). Assuming that errors are independent events, we can calculate the probability of a true base match, given the observed base match as

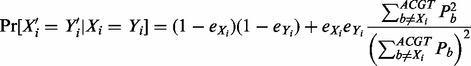

The probability of a true base match, given the observed base mismatch is

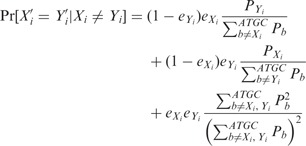

and the probability of a true base mismatch, given the observed base mismatch (or match) is

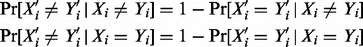

If any of the bases are undetermined (denoted by *N*),

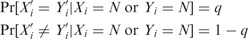



PEAR calculates the AS for each possible overlap [assuming no gaps, as they are infrequent on Illumina platforms (Nakamura *et al.*, 2011)] with a scoring matrix that rewards matches by a positive value α and penalizes mismatches with a negative value β. Scoring matrices for evaluating sequence alignments are being routinely used, for instance, in Basic Local Alignment Search Tool (Altschul and Gish, 1996) and Bowtie2 (Langmead and Salzberg, 2012). Empirical tests using simulated data showed that setting 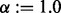 and 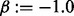 yield the best results (data not shown). Given the overlap 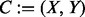, we define AS as

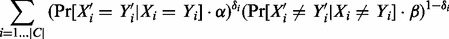

where

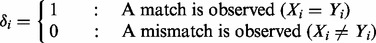

For the merged reads, PEAR computes the overlap that maximizes the AS. We denote the overlap that maximizes the AS by 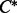.

### 2.2 Statistical test

To test the significance of the merged reads and to identify reads that shall not be merged, we calculate a *P*-value for the null hypothesis that the two corresponding reads are independent from each other. By independent we mean that any overlap between the two reads is purely by chance. For an overlap 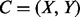 between two reads *x* and *y*, we define 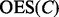 to be the *observed expected alignment score* (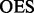)

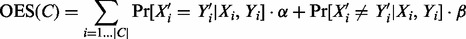

and

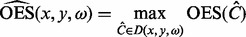

where 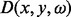 is the set of all possible overlaps between sequences *x* and *y* with a size of at least ω.

Let 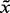 and 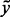 be two independent random sequences and let us further assume that there are no sequencing errors. Then, the *P*-value, that is, the probability of a random sequence producing an 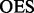 that is at least as high as the 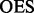 obtained from the merged reads. The *P*-value is defined as the probability of 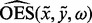 being greater or equal than the observed 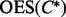. We obtain 


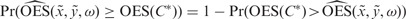
 


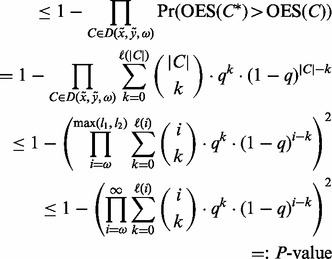

where

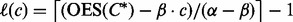

By default, PEAR uses an 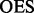 with a *P* 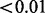 as cutoff. If the 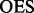 of the best merged read is smaller than this value, the reads will not be merged. Choosing a smaller *P*-value will reduce the FPR of the merged sequences, but a lower number of reads will be merged.

If the underlying overlap size is unknown, ω can be set to 1.0. If, however, the overlap is known to be short (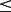
35-bp in our simulations), our statistical test will reject up to 4% (based on our simulations) correctly merged sequences because of low quality scores. To recover more merged reads, we provide the possibility to set ω to the computed overlap size *after* the merging step, instead of using a predefined fixed value. However, when using this work-around, the *P*-value of the statistical test is not valid anymore, as ω depends on the output of our algorithm. This implies that the random sequences are more restricted when choosing overlaps than the original input sequences. We will refer to the aforementioned, valid *P*-value as the *maximal accepted probability* (MAP). Our tests show that PEAR can produce 4% more merged sequences using MAP at the cost of a slight (∼0.1%) increase in FPRs.

### 2.3 Output

PEAR generates four FASTQ output files. One contains the successfully merged reads, two files contain the forward and reverse unmerged reads and one the discarded reads. Discarded reads are reads that fail to pass one of the following quality filters, which are applied after the merging process. These filters require the user to set some program parameters, which are outlined below.

**Minimal quality score for trimming**

It is common to trim the reads and use their high quality part due to the low quality of base calls toward the end of Illumina reads (Caporaso *et al.*, 2011). Consequently, PEAR includes the option to trim unmerged reads that contain at least two consecutive bases with quality scores lower than a user-specified *minimal quality score* value.

**Minimal length of output sequences**

PEAR discards merged sequences or trimmed unmerged reads that are shorter than this threshold.

**Maximal length of the output sequences**

PEAR discards sequences longer than the specified maximal length.

**Maximal proportion of uncalled bases**

This parameter allows for discarding reads that contain more than the specified proportion of uncalled bases N. When the value is set to 0, it will discard all reads containing one or more uncalled bases N.

Now, assume two reads *x* and *y can* be merged and have an overlapping region *C*. PEAR will correct errors in the overlapping region and compute updated quality scores for the overlap. For every pair of corresponding observed bases *X* and *Y* in *C* and their quality scores *e_X_* and *e_Y_*_,_, respectively, we distinguish four cases—*X* and *Y* are identical, different, one of them is uncalled or both of them are uncalled. When the two bases are identical, PEAR simply inserts this base into the corresponding position in the merged sequence and assigns the product of the quality scores: 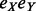 because errors are independent from each other (see Section 2.1). When the base pairs are different, PEAR inserts the base with the highest quality score and the corresponding quality score. If (only) one of the two bases is uncalled (N), PEAR uses the called base and its quality score. Finally, if both bases are uncalled, we arbitrarily use the lower of the two quality scores, as a quality score is required to obtain a valid FASTQ output file.

### 2.4 Parallelization and memory management

PEAR target platforms are off-the-shelf laptop and desktop computers. We implemented an appropriate memory allocator and manager that allows PEAR to use only a predefined amount of memory that the user can specify via a command-line switch. PEAR can use several gigabytes, but also just a few kilobytes of RAM.

Current off-the-shelf laptops and servers consist of multi-core processors with a minimum number of two cores per processor, thus increasing the total processing power of the system. However, RAM clock rates are still slower than CPU clock rates (also known as ‘memory-gap’). Thus, the time required for loading data from RAM into cache memories and registers can lead to performance deterioration.

To increase efficiency, PEAR takes into account both properties of modern hardware, that is, memory access patterns and the number of cores. Currently, most tools process sets of paired-end reads iteratively. They load a set of reads and merge them until all reads in this set have been merged. Because disk accesses are serial, most tools suffer from waiting times induced by loading reads into RAM and the caches. To alleviate this performance bottleneck, PEAR uses a standard double-buffering technique. The main idea of double-buffering techniques is to split-up the available RAM (specified by the user) into two buffers of equal size, which we denote as *active* and *passive* buffer. At program start-up there is an initial latency until the active buffer has been filled with reads for the first time. Then, a dedicated thread (which we denote as *reader* thread) loads a second set of reads into the passive buffer, whereas the remaining threads process the reads in the active buffer. If the reader thread has already loaded the next set while the remaining threads are still processing the reads in the active buffer, the reader thread will also start merging reads. In the opposite case, the remaining threads will idle until the reader thread has filled the passive buffer. Thus, to obtain ‘good’ parallel performance, the buffer size needs to be adapted to the system at hand (see later in the text).

Each thread only merges ∼500 reads from the active buffer at a time. This per-thread set size of 500 yielded the best performance in our experiments (data not shown)

When all reads in the active buffer have been processed, the first thread that finishes merging its assigned reads will become the reader. The reader swaps the active buffer with the passive buffer by simply flipping a memory pointer. Subsequently, it will start filling the new passive buffer with the next reads from the input file. The remaining threads will start processing reads from the new active buffer. Thereby, we can parallelize the process of reading from disk and merging reads. By using this technique, we hide the latency of disk accesses and can use the majority of threads/cores for merging short reads and thus reduce overall run time.

We observed that the optimal RAM setting on a 48 core Magny-Cours system (see Section 3.4 for details) is 200 MB. This is because each disk access only reads ∼100 MB, as we use two buffers of size 100 MB each. Thus, the disk access latency is overlapped by the remaining threads that work on the reads in the active buffer. On four cores of the same hardware configuration, the optimal empirical setting is 50 MB.

## 3 RESULTS AND DISCUSSION

To evaluate PEAR and compare it with the three state-of-the-art mergers (FLASH v1.2.6, PANDAseq v2.4, COPE v1.1.2), we used simulated datasets with varying overlap and DNA fragment sizes as well as the following two empirical datasets:

1. deep sequencing data of the *Staphylococcus aureus* genome by MacCallum *et al.* (2009),
2. reads generated from paired-end sequencing of a known single sequence (template) used by Masella *et al.* (2012) to test PANDAseq.

### 3.1 Simulated data

To mimic the sequencing of multiple hypervariable regions of 16S rRNA, we extracted a reference sequence dataset of 1000 full-length bacterial 16S rRNA gene sequences from the RDP classifier training dataset (Wang *et al.*, 2007). We then used ART (v1.5.0) (Huang *et al.*, 2012) to simulate 100-bp paired-end reads, with mean target DNA fragment sizes of 101, 150, 165, 180, 190 and 250-bp, and a standard deviation of 10-bp. We set the parameters of ART to generate target DNA fragments by randomly sampling the reference sequences until a 10-fold coverage of the reference dataset was reached. To obtain a more realistic test dataset, we used two read quality profiles for simulating either end of the respective pairs. The target DNA fragments produced by ART provide the ground truth for the merged paired-end reads.

We also generated an additional set of 150-bp long reads with a mean fragment size of 101-bp, by extending all single reads in the above 101-bp fragment size set to a length of 150-bp. We extended the reads by complete random sequences with the lowest possible quality scores. This setup emulates case C (see Fig. 1) where the DNA fragment size is smaller than the length of a single-end read.

We executed PEAR, COPE, FLASH and PANDAseq on the above datasets and compared the lengths of the merged reads with the true fragment lengths. We only consider merged sequences whose length is equal to the true fragments size as correct merged sequences. When the fragment size is at least twice as long as the single read length, we consider that a result returning unmerged reads is correct. We executed PEAR with three different settings: (i) statistical test disabled, (ii) 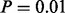 and (iii) MAP = 0.01. In all tests the minimum overlap size is 1; for all other parameters we use the default values. We ran PANDAseq with default parameters as well as with a minimal overlap setting of 10-bp. FLASH requires the mean fragment length and a proper minimal overlap value to work correctly. Therefore, we ran it with the known/true mean fragment lengths. COPE includes four different modes of execution. Mode 0 is similar to the FLASH approach, but with more stringent alignment score parameters. Modes 1 and 2 further use *k*-mer frequencies, and full-mode runs all three modes sequentially and concatenates the results. COPE generated a segmentation fault on our simulated data under COPE modes 1 and 2. Therefore, we only report results obtained under COPE mode 0.

Table 1 shows experimental results. With the exception of the first test case (no overlaps), PEAR consistently generates a larger number of correctly merged sequences when the statistical test is disabled. PEAR merges fewer correct fragments when the statistical test is enabled. When setting the *P*-value or MAP to 0.01, PEAR shows lower FPRs than all three competing mergers. When we use MAP to evaluate the merged reads, PEAR produces more merged reads with an FPR that is analogous to the FPR generated by PEAR *with* the statistical test. PEAR is robust with respect to short overlaps because it can still merge ∼40% of the reads when the mean overlap is only 10-bp. The FPR of 0.64% (MAP = 0.01) under this setting is 10 times lower than for FLASH and PANDAseq. When reads do not overlap, PEAR classifies them as unmerged with an FPR of 0.03%. Here, the FPR is defined as the fraction of merged reads that should not have been merged. Overall, PEAR shows low FPRs across all test scenarios (overlap lengths). In addition, it does not require any prior knowledge regarding overlap lengths. Therefore, PEAR can be used for merging sequences with varying fragment sizes. PANDAseq performs equally well as PEAR for the majority of cases where the overlaps exceed 20-bp. However, its FPR increases with decreasing overlap size, regardless of the minimal overlap size setting. Furthermore, PANDAseq incorrectly merges 55.4% of the reads that do not overlap and 25.11% of the reads when the mean overlap is set to 10-bp. We will discuss the reasons for this behavior in Section 3.5.

**Table 1.** Simulated dataset of paired-end reads with different overlap sizes

Software	Merged	Correct (−)	Correct (%)	FPR (%)
100-bp paired-end reads with no overlaps (23 096 pairs)				
COPE (mode0)	31	23 065	99.87	0.13
FLASH	0	23 096	100	0
PANDAseq (default)	12 796	10 300	44.59	55.4
PANDAseq (−o = 10)	10 562	12 534	54.27	45.7
PEAR (test disabled)	8 184	14 912	64.57	35.4
PEAR (*P* = 0.01)	8	23 088	99.96	0.03
PEAR (MAP = 0.01)	33	23 063	99.86	0.14
100-bp paired-end reads with 10-bp mean overlaps (24 969 pairs)				
COPE (mode0)	5 755	5 709	22.86	0.80
FLASH	8 968	8 309	33.27	7.34
PANDAseq (default)	19 616	14 690	58.83	25.11
PANDAseq (−o = 10)	17 783	12 053	48.27	32.22
PEAR (test disabled)	19 691	17 112	68.53	13.10
PEAR (*P* = 0.01)	9 365	9 315	37.31	0.53
PEAR (MAP = 0.01)	10 080	10 015	40.11	0.64
100-bp paired-end reads with 20-bp mean overlaps (25 858 pairs)				
COPE(mode0)	9 819	9 750	37.71	0.70
FLASH	10 917	10 843	41.93	0.67
PANDAseq (default)	23 136	21 596	83.51	6.65
PANDAseq (−o = 10)	22 736	20 722	80.14	8.85
PEAR (test disabled)	24 153	23 386	90.44	3.16
PEAR (*P* = 0.01)	18 202	18 115	70.06	0.48
PEAR (MAP = 0.01)	19 265	19 165	74.12	0.52
100-bp paired-end reads with 35-bp mean overlaps (27 026 pairs)				
COPE (mode0)	11 771	11 693	43.27	0.66
FLASH	15 603	15 507	57.37	0.61
PANDAseq (default)	26 068	25 849	95.64	0.84
PANDAseq (−o = 10)	26 267	26 026	96.29	0.92
PEAR(test disabled)	26 866	26 712	98.84	0.57
PEAR (*P* = 0.01)	25 939	25 833	95.59	0.41
PEAR (MAP = 0.01)	26 380	26 273	97.21	0.41
100-bp paired-end reads with 50-bp mean overlaps (28 339 pairs)				
COPE (mode0)	7 915	7 858	27.73	0.72
FLASH	20 025	19 940	70.36	0.42
PANDAseq (default)	27 939	27 834	98.21	0.37
PANDAseq (−o = 10)	28 049	27 944	98.61	0.37
PEAR(test disabled)	28 335	28 234	99.63	0.36
PEAR (*P* = 0.01)	28 288	28 190	99.47	0.35
PEAR (MAP = 0.01)	28 329	28 229	99.61	0.35
150-bp paired-end reads with 100-bp mean overlaps (33 217 pairs)				
COPE (mode0)	43	0	0	100
FLASH	44	0	0	100
PANDAseq (default)	11 417	0	0	100
PANDAseq (−o = 10)	14 146	0	0	100
PEAR (test disabled)	33 187	33 071	99.56	0.35
PEAR (*P* = 0.01)	33 136	33 022	99.41	0.34
PEAR (MAP = 0.01)	33 185	33 071	99.56	0.34

FLASH failed to merge the majority of reads for small overlap sizes, but exhibits low FPRs for merged sequences. FLASH merges reads by maximizing the fraction *f* (number of matches to overlap size ratio). The default threshold of *f* in FLASH is 0.75 and the default minimal overlap size (ω) is 10. This setting can be shown to have a *P*-value of 0.00156 for merged reads by using the statistical test introduced in Section 2.2 and replacing 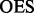 with *f*. However, overlaps that exclusively maximize *f* might not yield correctly merged sequences. Let us consider two possible overlap sizes 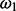 and 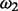 for paired-end reads *x* and *y*, where 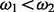. As an example, we assume 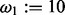 with 1 mismatch, 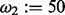 with 6 mismatches and a true overlap size of 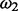. Then 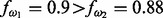 and FLASH will choose the overlap of size 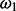 as merged read. Because 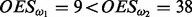, PEAR will return the correct result. FLASH also requires the mean fragment length as input, which restrains its applicability to datasets with uniform fragment length.

COPE, PANDAseq and FLASH were unable to merge reads under application scenario C (see Fig. 1) where the DNA fragment size is smaller than a single-end read (Table 1). PANDAseq incorrectly merges over one-third of the reads in this scenario.

### 3.2 *S.aureus* genome data

This dataset was initially generated by MacCallum *et al.* (2009) (available for download at http://gage.cbcb.umd.edu/data) to assess short read-based genome assembly quality. We used the raw dataset that contains 647 052 pairs of 101-bp long reads with a mean DNA fragment size of 180-bp and 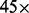 coverage of the *S.aureus* genome. To determine the true target DNA fragment sizes, we used Bowtie2 (Langmead and Salzberg, 2012) to map the merged reads to the reference genome. We use the corresponding *end-to-end mode* in Bowtie2 and do not allow for opening gaps in either sequence (the reads *and* the reference genome). This guarantees that all merged reads that can be mapped to the reference genome are correctly merged. This is because there are two possible scenarios for incorrectly merged reads: (i) they can be longer than the corrected one, in which case the sequences can be aligned by opening gaps in the reference sequence or (ii) they are shorter than the corrected one, and the sequences can be aligned by opening gaps in the merged sequences. Therefore, we consider that a merged paired-end read is correct only if Bowtie2 finds a hit on the reference genome. Note that, the results are conservative because some of the correctly merged reads might be missed by Bowtie2 due to sequencing errors.

We summarize the results in Table 2. All mergers work well in this setting. PANDAseq correctly merges the highest amount of reads; PEAR ∼2% less (statistical test disabled). Nonetheless, a quarter of the reads merged by PANDAseq were not mapped to the reference genome using Bowtie2. In contrast only 4.9% of the merged reads from PEAR could not be mapped. COPE merges less reads than PEAR and shows a lower FPR when the statistical test in PEAR is disabled. This is likely because COPE is specifically designed for such deep sequencing datasets.

**Table 2.** A total of 647 052 paired-end reads with mean fragment size 180-bp and read length 101-bp (*S.aureus* genome)

Software	Merged	Correct (−)	Correct (%)	FPR (%)
COPE (full mode)	373 543	369 683	57.13	1.03
FLASH	369 276	361 663	55.89	2.06
PANDAseq (default)	534 839	418 747	64.72	21.71
PANDAseq (−o = 10)	533 618	407 477	62.97	23.64
PEAR (test disabled)	411 321	391 157	60.45	4.90
PEAR (*P* = 0.01)	202 221	199 764	30.87	1.22
PEAR (MAP = 0.01)	257 409	251 714	38.90	2.21

### 3.3 Single known sequence data

We used a dataset that was deployed by Masella *et al.* (2012) to assess PANDAseq. The dataset contains paired-end reads from a single template sequence. The template sequence is the V3 region of the *Methylococcus capsulatus* (ATCC 33009) 16S rRNA gene. It has a length of 198-bp, including the primers. The FASTQ files contain 673 845 pairs of 108-bp long paired-end reads. Each pair overlaps by exactly 18-bp. We calculate the ‘true’ merged reads by computing a global pairwise sequence alignment between the merged reads and the template sequence. Subsequently, we check if the overlapping region contains gaps, we consider a merged read to be correct if there is no gap. We also calculate the ER of the merged reads to evaluate error correction performance. The ER is the average number of errors per merged read (excluding gaps) with respect to the template sequence. We ran PEAR with default parameters. We executed PANDAseq with default parameters and with a minimum overlap setting of 10-bp. We applied FLASH with a template sequence length of 198-bp and a read length of 108-bp.

For this dataset, PEAR merges the highest number of reads when the statistical test is disabled (Table 3). When setting 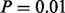 and using the test, less reads are merged, but only 0.03% of the merged reads are false positives. Both, PANDAseq and FLASH, produce comparable results but with a slightly higher FPR. We executed COPE in full mode (see Section 3.1) on this dataset. COPE did not merge any reads, however. The ER of the raw reads is 0.51. Although the overlap size is only 18-bp all mergers decrease the ER. Merged reads produced by FLASH and PANDAseq show ERs that are slightly lower than PEAR (statistical test disabled). However, PEAR yields three times lower ERs when the statistical test is enabled.

**Table 3.** Single template 198-bp sequence dataset of 673 845 108-bp paired-end reads

Software	Merged	Correct (−)	FPR (%)	ER
COPE (full mode)	0	0		
FLASH	660 984	660 030	0.14	0.4594
PANDAseq (default)	660 593	657 602	0.45	0.4333
PANDAseq (−o = 10)	660 522	657 609	0.44	0.4304
PEAR (test disabled)	663 025	661 717	0.20	0.4753
PEAR (*P* = 0.01)	576 225	576 035	0.03	0.1470
PEAR (MAP = 0.01)	578 887	578 679	0.04	0.1486

### 3.4 Run time and memory requirement

**Fig. 2.**
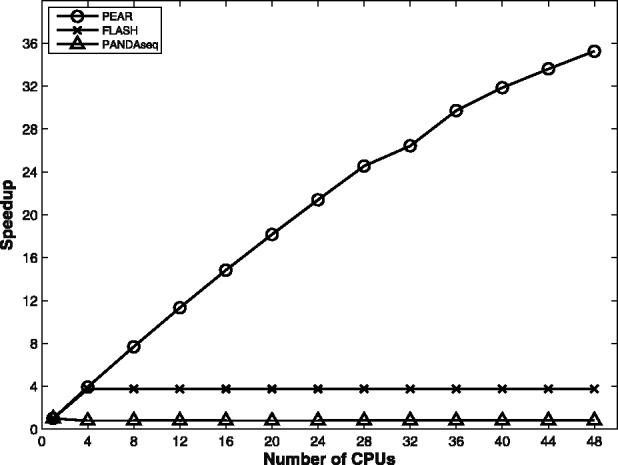
Parallel speedups of PEAR, FLASH and PANDAseq on the single template sequences dataset. The sequential runtimes for the three mergers are 98, 58 and 39 s, respectively

To compare run times between PEAR and competing mergers, we used the dataset from Section 3.3. We conducted experiments on an AMD Opteron 6174 2.2 GHz 4-processor machine with 12 cores each and a total of 48 cores. We used the default PEAR memory setting of 200 MB. PANDAseq is the fastest tool on a single core. Surprisingly, when running the experiment with four cores, which corresponds to a standard desktop PC, PANDAseq is slower than for the sequential run, whereas PEAR and FLASH are equally fast and twice as fast as PANDAseq. However, the main observation is that neither PANDAseq nor FLASH yields substantial speedups when executed on several cores (see Fig. 2). In fact, although PEAR yields linear speedups, FLASH and PANDAseq yield no speedup at all. We excluded COPE from most experiments because it does not merge any reads for this dataset and because it has only been partially parallelized; only the *k*-mer computation is parallelized. For the sake of completeness, the overall run time of COPE on this dataset is 395 s using the parallel *k*-mer computation on 48 cores (minimum memory requirement of 16 GB). Hence, COPE is substantially slower than the other programs we tested.

We also tested PEAR, FLASH and PANDAseq on a substantially larger dataset of 36 504 800 101-bp long paired-end reads from the human chromosome 14 (data available at http://gage.cbcb.umd.edu/data). Using 48 cores, PEAR only requires 62 s to finish, whereas FLASH and PANDAseq need 7 and 21.5 min, respectively.

### 3.5 Reasons for high FPRs in PANDASeq

PANDAseq merges reads by choosing the overlap *C*, such that 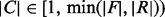 that maximizes
(1)
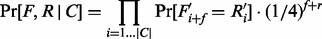

where *F* is the forward read sequence and *R* is the reverse read sequence. When the DNA fragment size exceeds the sum of the lengths of the reads (see Fig. 1, case B), a merger should not merge the reads. According to Equation 1, PANDAseq will merge reads with an overlap *C*, when
(2)
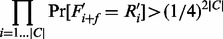

Assuming that the merged sequences are generated randomly with all base frequencies being equally likely with probability 0.25 and that all bases have an equal error probability *e*, we can simplify Equation (2) to

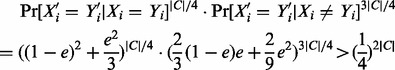

Solving the above inequality, we obtain 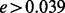. In other words, when the bases have an average error probability that is >0.039, PANDAseq will favor merging randomly generated sequences. Because the quality of Illumina reads decreases toward the end of the reads, PANDAseq will therefore incorrectly merge reads that do not overlap.

## 4 CONCLUSIONS

We introduce PEAR, a new software tool that produces highly accurate merged Illumina paired-end reads with low FPRs. It can merge paired-end read datasets under settings where most competing mergers fail. Furthermore, PEAR does not require preprocessing or quality control before merging. One main application scenario is the merging of paired-end reads from datasets with varying DNA fragment sizes. We have also introduced a statistical test to evaluate the merged read. Finally, PEAR scales well on most server and desktop computers. We intend to implement an automatic buffer size tuning routine in PEAR to maximize performance without user intervention.

*Funding*: J.Z. and K.K. are funded by a HITS scholarship. T.F. is funded by DFG project STA-860/4. The authors acknowledge the support of Graduate School for Computing in Medicine and Life Sciences, University of Lübeck.

*Conflicts of Interest*: none declared.
